# Exploring the impact of digital health literacy on quality of life in patients undergoing retrograde intrarenal surgery for kidney stone treatment: a prospective, single-center study

**DOI:** 10.1007/s00240-024-01576-1

**Published:** 2024-05-23

**Authors:** Ahmet Keles, Ozgur Arikan, İlkin Hamid-zada, Umit Furkan Somun, Kursad Nuri Baydili, Asif Yildirim

**Affiliations:** 1 Department of Urology, School of Medicine, Istanbul Medeniyet University, Istanbul, Turkey; 2 Department of Biostatistics, School of Medicine, University of Health Sciences, Istanbul, Turkey

**Keywords:** Digital, Health, Literacy, Patient, Reported, Outcome, Kidney stone, Quality of life

## Abstract

Retrograde intrarenal surgery (RIRS) is the recommended treatment for renal stones up to two cm in size. As digital health literacy (e-HL) has become increasingly important in promoting informed health decisions and healthy behaviors, it is necessary to investigate its impact on RIRS treatment outcomes. We aimed to explore the influence of patients’ e-HL level on their postoperative quality of life (QoL). We conducted an observational prospective study of 111 patients who underwent RIRS for renal pelvis stones. Before RIRS, we evaluated patients’ e-HL using the electronic health literacy scale (eHEALS). QoL was evaluated using the five-level EuroQol five-dimensional questionnaire (EQ-5D-5L) one month after RIRS. SFR was determined by a negative CT scan or asymptomatic patients with stone fragments < 3 mm. Adult individuals aged 18 years or older with typical calyceal anatomy met the eligibility criteria for enrollment. Exclusion criteria for the study included patients with ureteric stones, anomalous kidneys, or bilateral renal stones. The relationship between patients’ QoL and stone-free rate was explored using Spearman’s rank correlation coefficient. The mean stone burden was 14 ± 3 mm (6–19 mm). The overall SFR was 83.3% after one month. The median EQ-5D-5L utility index and VAS score were 0.826 (0.41–1) and 70 (20–100) respectively, for the overall population. We found that poorer e-HL was associated with being older (p = 0.035), having less education (p = 0.005), and not having access to the internet (p < 0.001). A significant difference was observed between patients with sufficient e-HL and patients with limited e-HL in the self-care (p = 0.02) and anxiety/depression (p = 0.021) dimensions. To date, no study has examined the impact of patients’ e-HL levels on postoperative QoL in patients undergoing RIRS. This study also revealed that e-HL levels in patients undergoing RIRS were related to postoperative QoL, especially self-care and anxiety/depression dimensions, whereas there was no relationship between them and SFR.

## Introduction

Globally, the incidences and recurrence rates of kidney stones are, resulting in a significant increase in treatment costs and substantial health challenge [1, 2]. Meanwhile, improvements in endoscopy technology have made the RIRS an attractive treatment option for renal stones. In recent years, many studies have focused on RIRS to be a safe technique, and it is associated with minimal and minor complications for intrarenal stone [3]. Previous research has found that most patients search for health-related information online [4]. Since COVID-19, Internet technology and electronic resources have become increasingly critical in everyday life. Social media also play a significant role in offering communication channels and providing emotional support for patients with cancer [7]. With increasing usage and reliable health information, the Internet is also filled with misleading information. This creates a major challenge for cancer patients and affects their confidence in online medical information. That is why patients must have a certain level of ability to interpret and deal with online health information from the Internet. The idea of digital health literacy (e-HL) encapsulates these abilities [8].

We hypothesize that providing kidney stone patients with information on disease-related processes, risks, recommendations, and possible situations that may occur after RIRS could play a critical role in assisting them in coping with the difficulties they experience and improving their QoL. However, no studies have yet explored the connection between e-HL, and QoL reported by patients undergoing treatment for kidney stones with RIRS. We conducted a study at a single institution to analyze the relationship between patient-reported QoL outcomes after RIRS and e-HL in patients with renal pelvis stones.

## Materials and methods

### Design and ethical principles of the study

After obtaining ethical approval from the Istanbul Medeniyet University School of Medicine Institutional Review Board (Number:2023/0057, Date: 25.01.2023), we conducted a prospective, nonrandomized cohort study from May to September 2023 at a tertiary university hospital, which serves as a reference center for stone disease.

### Sample selection and data collection

The study focused on patients eligible for inclusion, specifically those who underwent RIRS at an age less than 80 years, with a tone size set at a maximum of 3 cm. Patient characteristics, including age, sex, number of stones, cumulative stone diameter, stone location, and hydronephrosis grade, were recorded. CT scans were used for preoperative and postoperative evaluations. Before the surgery, all patients needed to undergo a sterile urine culture test. Exclusion criteria included bilateral renal stones, ureteral stones, urinary tract infections, multi-stage procedures, and other abnormalities such as solitary kidney and ureteral strictures.

### Defining the instruments and measurement

The evaluation of e-HL utilized the Turkish adaptation of the eHealth Literacy Scale (eHEALS) created by Norman and Skinner in 2006. This tool gauges various aspects of literacy, including traditional, health-related, information retrieval, scientific research, media, and computer literacy (13). Each factor is scored on a 5-point Likert scale ranging from 1 (strongly disagree) to 5 (strongly agree). The score ranges from 8 to 40, indicating the aptitude for using e-health information for health decisions. For each patient, the scores for each question were summed to obtain an e-HL score. Stone-free status was defined as no evidence of stone fragments or the presence of nonsymptomatic residual fragments less than 3 mm, evaluated with non-contrast computed tomography in the first month after surgery.

The EQ-5D-5L questionnaire was used to assess patient quality of life by measuring health-related status in five dimensions: mobility, personal care, usual activities, pain/discomfort, and anxiety/depression. The questionnaire consists of five levels of severity for each dimension, and higher values indicate a better quality of life. Additionally, the EQ-VAS part of the questionnaire was used to measure health status on a scale from 0 to 100, with higher scores indicating better health status [11]. A unique five-digit code can refer to an EQ-5D health state (e.g., 11,111 = no problems in any area) or a single EQ-5D utility index score can refer to a health state worse than death. All interviews were conducted face-to-face by the urologist at 8 weeks after during the post-operative visit.

### Procedure description

All RIRS procedures were conducted under general anesthesia and performed by experienced urologists specializing in minimally invasive procedures. Prophylactic antibiotics were administered intravenously two hours before the procedure. A 21 F cystoscope was inserted to visualize the ureteral orifice and a hydrophilic guide wire was inserted. For mechanical dilatation, a 9.8 F semi-rigid ureteroscope was used that reached the ureteropelvic junction. Subsequently, a ureteral access sheath of varying sizes (9.5/11.5 Fr, Plastimed, Turkey) was placed, enabling the surgeon to navigate the flexible ureteroscope (Flex X2^®^, Karl Storz, GmbH, Germany) into the renal collecting system. Stones were identified using holmium laser with a 275 µm fiber and fragmented using laser energy settings configured at 0.8–1.2 J with a frequency of 8–12 Hz. A “stone-free” status was determined by the lack of any visible residual stones or the presence of fragments no larger than 3 mm on a CT scan taken one month after the initial surgery. To decrease the likelihood of ureteric obstruction and renal colic following ureteral edema, a Double J stent was routinely inserted and removed approximately two weeks after the operation.

### Statistical analysis

Data analysis was conducted using the IBM SPSS 25 software package. Frequency and percentage values are presented for categorical variables. The normality of the quantitative variables was tested using the Shapiro–Wilk test. For quantitative variables, median, minimum, and maximum values were provided. The Mann–Whitney U test was used for comparisons between two categorical qualitative variables and quantitative variables, whereas the Kruskal–Wallis H test was used for comparisons between more than two categorical qualitative variables and quantitative variables. In case of a significant difference found in the Kruskal–Wallis H test, categories were compared pairwise using the Bonferroni-corrected Mann–Whitney U test. The chi-squared test was used to compare two categorical variables. The presence of a relationship between the two quantitative variables was examined using Spearman’s correlation. In this study, a Type I error rate of 0.05 was adopted.

## Result

### Demographic characteristics of study participants

**Fig. 1:**
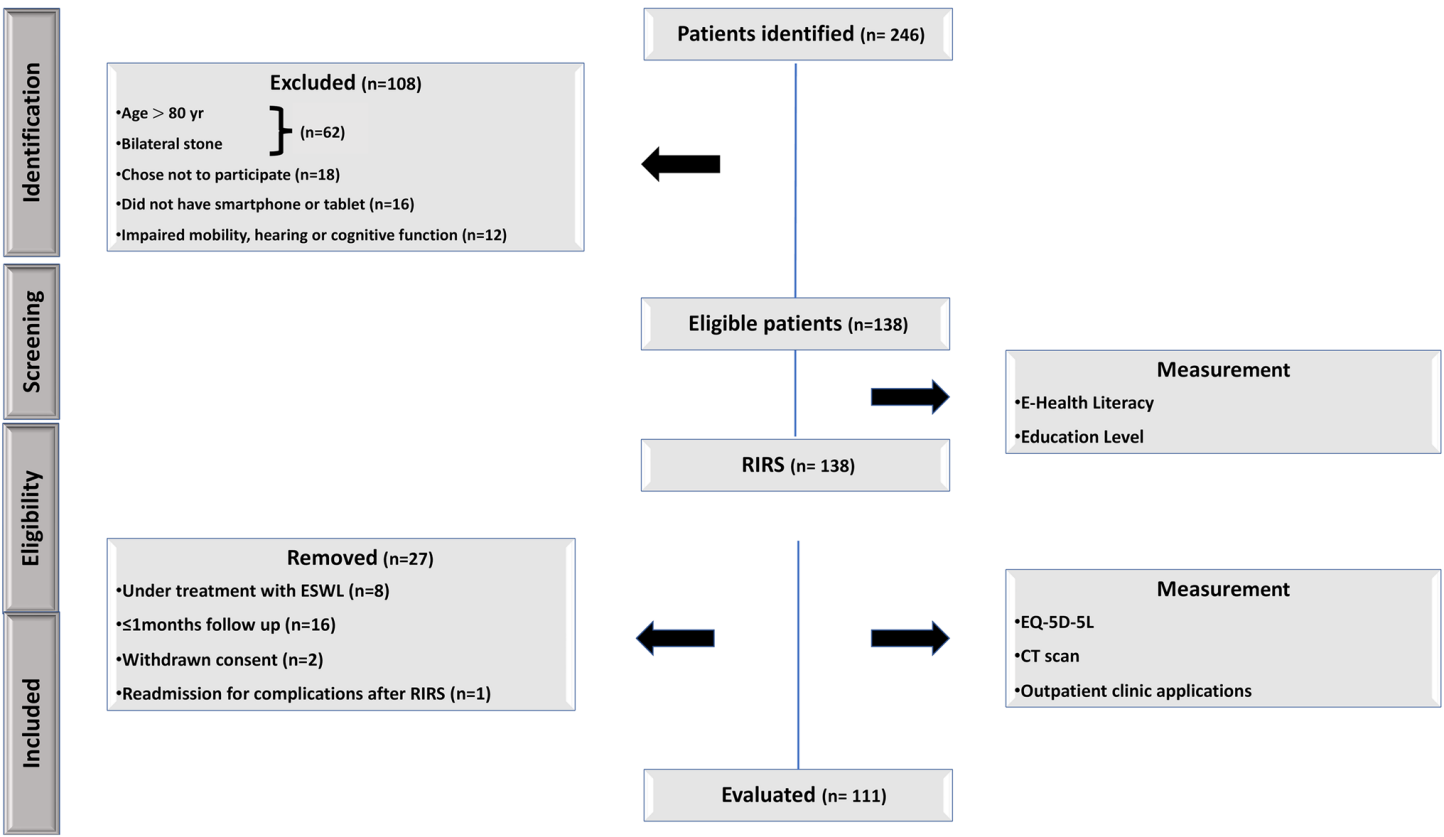
An overview of the study's participant tracking flowchart

A total of 238 individuals were screened for eligibility criteria, of which 111 met the requirements and were enrolled in the study. The sex distribution was 62 males (64.9%) and 49 females (35.1%). The flow chart of the study is shown in Fig. 1. Moreover, the median patient age and BMI at surgery were 52 years (range, 27–76) and 26.3 kg/m^2^ (range, 14.8–31.4), respectively. Most of the respondents had elementary education (40.4%) and were married (74.8%). Table 1 shows demographic, clinical of the participants’ demographic information.

**Table 1:** The analyses to identify the factors that impact digital health literacy scores

Variables		N = 111 (%)	eHEALS Score			Differences
			Median (min–max)		p value*	
Age (yr)	< 50 (A)	54 (48.6)	30 (12–35)	−2.104^U^	0.035*	A > B
	≥ 50 (B)	57 (51.3)	24 (11–40)			
Gender	Male	62 (64.9)	25 (12–40)	−0.722^U^	0.470	–
	Female	49 (35.1)	28 (11–37)			
BMI (kg/m^2^)	Normal (< 25)	29 (26.1)	27.5 (15–35)	1.682^H^	0.431	–
	Overweight (25–30)	56 (50.4)	27 (12–40)			
	Obese (≥ 30)	26 (23.4)	24 (11–36)			
Education level (ISCED level)	Level 1	45 (40.5)	24 (11–40)	12.919^H^	0.005*	D > A, B
	Level 2	14 (12.6)	24 (18–35)			
	Level 3	24 (21.6)	27.5 (12–32)			
	Level 4	28 (25.2)	32 (12–39)			
Martial status	Single	28 (25.2)	25 (11–36)	−1.683^U^	0.092	–
	Married	83 (74.8)	27 (15–40)			
Internet usage	Almost everyday	49 (44.1)	30.5 (16–39)	23.307^H^	< 0.001*	A > B, C, D, E
	Few days a week	36 (32.4)	24 (12–40)			
	Less than 1 day a week	15 (13.5)	23 (11–33)			
	Hardly ever	11 (9.9)	19 (16–24)			

*Indicates statistical significance

^U^, The Mann–Whitney U test statistic; ^H^, The Kruskal–Wallis H test statistic; yr, years; BMI, Body mass index; ISCED, International Standard Classification of Education

### Operative characteristics and postoperative outcomes

The mean stone size was 14 ± 3 mm (6–19 mm), and an access sheath was used in 90.6% of patients. The mean operation time was 63.7 ± 11.4 min. The mean stone-free rate was 83.3% in all patients. In our sample, the stone-free rate was not correlated with the e-HL scores (p = 0.013). In the short-term follow-up at two months, no serious complications (Clavien III-V) were observed in any of the patients. Moreover, e-HL was not associated with post-discharge outcomes, namely 30 day emergency clinic application or 90 day hospital readmissions.

### Factors influencing e-HL

The examination of the factors influencing e-HL underscores the nuanced relationship between certain demographic variables and e-HL. The investigation of the relationship between e-HL scores and various demographic factors revealed notable patterns. As age increased, e-HL scores exhibited a discernible decrease, indicating a potential decline in e-HL proficiency among older individuals (p = 0.008). Furthermore, a statistically significant positive association was observed between the e-HL scores and higher education levels (p = 0.005). In subgroup analyses, it was found that the ratio of people with a primary school education or lower was higher in the limited e-HL group (p < 0.001). An interesting trend emerged when examining the impact of annual income on e-HL scores. (p = 0.013). Similarly, participants within affluent annual income brackets tended to have higher e-HL scores. Moreover, respondents who reported higher levels of Internet usage demonstrated elevated e-HL scores, indicating a positive relationship between digital engagement and e-HL (p < 0.001). The e-HL group showed clinically insignificant differences in the length of hospital stay.

### Quality of life assessment

Table 2 illustrates the correlation between the eHEALS score and EQ-5D-5L index, VAS, and RIRS scores. We found that eHEALS scores were significantly associated with EQ-5D-5L index scores (r = 0.236, p = 0.013). There was no relationship between postoperative EQ‐5D‐5L index values and the patient's preoperative evaluated RIRS score (r = 0.236, p = 0.013). Notably, patients characterized by EQ-VAS status demonstrated no significant differences in the EQ-5D-5L index score (r = 0.15, p = 0.117). When comparing the specific dimensions of the EQ-5D-5L questionnaire based on e-HL levels, there were statistically significant differences in self-care (p = 0.02) and anxiety/depression (p = 0.021) between patients with adequate and limited e-HL. However, no significant differences were found in the e-HL levels for mobility, usual activities, and pain/discomfort (Table 3).

**Table 2:** Spearman correlation coefficients between EQ-5D-5L dimensions and eHEALS, VAS, and RIRS scores

Variables		Patients’ digital health literacy
		eHEALS Score
EQ-5D-5L	rho	0.236
	p	0.013*
EQ-VAS	rho	0.15
	p	0.117
RIRS Score	rho	−0.046
	p	0.630

*Indicates statistical significance

Analysis done with spearman correlation

EQ-5D-5L, Five-level EQ-5D, Higher values indicate better QoL, EQ-VAS, EuroQol-Visual Analog Scale, E-HEALS, e-health literacy scale, 1 (strongly disagree) to 5 (strongly agree), with total e-health literacy scores ranging from 8 (lowest possible e-health literacy) to 40 (highest possible eHealth literacy), VAS, Visual analog scale, RIRS, Retrograde intrarenal surgery

**Table 3:** Distribution and comparison of EQ-5D-5L dimensions outcomes stratified by digital health literacy score

EQ-5D-5L		Limited e-HL n = 51, (%)	Adequate e-HL n = 60, (%)	Chi-squared	p
Mobility	No problems	31 (60.8)	48 (80)	5.133	0.077
	Slight problems	17 (33.3)	11 (18.3)		
	Moderate problems	3 (5.9)	1 (1.7)		
	Severe problems	–	–		
	Unable to	–	–		
Self-care	No problems	20 (39.2)	39 (65)	8.831	0.02*
	Slight problems	24 (47.1)	19 (31.7)		
	Moderate problems	6 (11.8)	2 (3.3)		
	Severe problems	1 (2)	–		
	Unable to	–	–		
Usual activities	No problems	27 (52.9)	36 (60)	1.958	0.665
	Slight problems	18 (35.3)	19 (31.7)		
	Moderate problems	6 (11.8)	4 (6.7)		
	Severe problems	–	–		
	Unable to	–	1 (1.7)		
Pain/discomfort	No problems	11 (21.6)	19 (31.7)	3.637	0.417
	Slight problems	24 (47.1)	28 (46.7)		
	Moderate problems	14 (27.5)	13 (21.7)		
	Severe problems	1 (2)	–		
	Unable to	1 (2)	–		
Anxiety/depression	No problems	23 (45.1)	42 (70)	7.674	0.021*
	Slight problems	22 (43.1)	16 (26.7)		
	Moderate problems	6 (11.8)	2 (3.3)		
	Severe problems	–	–		
	Unable to	–	–		

*Indicates statistical significance

Higher values indicate better QoL

EQ-5D-5L, Five-level EQ-5D; e-HL, Digital health literacy

## Discussion

Increasing life expectancy and the development of medical technology have raised people's awareness of their QoL. This study was the first to investigate the influence of e-HL on QoL in patients who underwent RIRS for kidney stones. E-HL has demonstrated efficacy in improving outcomes in chronic diseases; nevertheless, its impact on surgical outcomes and quality of life remains inadequately documented [11, 12]. We chose to examine the kidney stone population because kidney stones have many treatment options, ranging from surgery to active surveillance. Hence, patients with kidney stones are expected to assume personal responsibility for their health, make knowledgeable decisions despite intricate health matters, and maneuver a convoluted healthcare system. Therefore, adequate e-HL is crucial.

E-HL scores showed a visible decline pattern with advancing age in our study, as in other studies; this suggests that younger individuals tend to show higher levels of e-HL [13]. This age-related trend may be indicative of the digital divide, where younger generations, having grown up in an era dominated by technology, are more adept at navigating and utilizing online health information. Conversely, we also found a positive relationship between e-HL scores and higher education levels, meaning that individuals with more educational backgrounds are better equipped to understand and critically evaluate health-related information available on the Internet [13]. Similar findings were observed in studies by Wright et al. and Luckenbaugh [14, 15]. Moreover, the significant correlation between affluent annual income brackets and elevated e-HL scores underscores the role of socioeconomic factors in shaping e-HL, potentially limiting access and proficiency for those in lower income groups [16]. Additionally, the positive link between Internet usage and higher e-HL scores underscores the importance of familiarity with and comfort with online platforms in enhancing e-HL. By considering these factors, interventions can be tailored to the specific needs of different demographic groups, ultimately promoting equitable access to reliable online health information.

Our findings support the results reported by Zheng et al. and Berkman et al., who described health literacy as moderately correlated with QoL [17, 18]. Our analysis revealed statistically significant differences in EQ-5D-5L scores among the different e-HL groups, particularly in the dimensions of anxiety and depression. This suggests that individuals with lower e-HL may experience higher levels of anxiety and depression, which can negatively impact their overall QoL [13].

Our study provides insights into the effect of e-HL on QoL in patients with kidney stones who underwent RIRS in a real-world setting. It is important to note, however, that this study had some limitations. In the first place, the sample size was small, so further research with larger samples is needed to confirm the findings. A further risk of bias is associated with some data that are based on self-reported questionnaires. As a final point, we did not include any specific questionnaire to measure patient satisfaction with stenting. Additional research is necessary to gain a more comprehensive understanding of how e-HL affects QoL and psychological well-being, as well as its true impact on these aspects.

## Conclusion

In conclusion, the findings of this study highlight the importance of e-HL in influencing the QoL of patients who have undergone RIRS for kidney stones. The results emphasize the need for adequate e-HL in empowering patients to make informed decisions and navigate the complexities of healthcare systems, particularly in the context of managing kidney stones. This study also contributes to filling the gap in understanding the impact of e-HL on surgical outcomes, shedding light on its significance in the overall well-being of patients with chronic medical conditions. The prospective and observational nature of this study adds valuable insights into the preference-weighted QoL data of kidney stone patients undergoing RIRS, paving the way for further research and interventions aimed at improving e-HL and QoL in this patient population.

## Data Availability

The data supporting the findings of this study are not openly available owing to sensitivity concerns and can be obtained from the corresponding author upon reasonable request. The data were stored in controlled access data storage at the Istanbul Medeniyet University School of Medicine, Goztepe Prof. Dr. Suleyman Yalcın City Hospital.
